# Astilbin reduces ROS accumulation and VEGF expression through Nrf2 in psoriasis-like skin disease

**DOI:** 10.1186/s40659-019-0255-2

**Published:** 2019-09-06

**Authors:** Wuyuntana Wang, Yuhai, Huan Wang, Chasuna, Bagenna

**Affiliations:** 1 Department of Mongolian Pharmacy, School of Mongol Medicine, Inner Mongolia University for Nationalities, 536 West of Huolinhe Street, Tongliao, 028000 Inner Mongolia People’s Republic of China; 2 Department of Dermatology, Affiliated Hospital of Inner Mongolia University for Nationalities, Tongliao, Inner Mongolia People’s Republic of China; 3 Library of Inner, Mongolia University for Nationalities, Tongliao, Inner Mongolia People’s Republic of China

**Keywords:** Astilbin, ROS, Nrf2, Psoriasis

## Abstract

**Background:**

Psoriasis is a common and intractable skin disease affecting the physical and mental health of patients. The accumulation of ROS is involved in the pathogenesis of psoriasis and antioxidants are believed to be therapeutic. This study aimed to investigate the therapeutic efficacy of astilbin on ROS accumulation in psoriasis.

**Results:**

The study showed that 50 μg/ml astilbin could inhibit the growth and reduce the accumulation of ROS in HaCaT cells stimulated by IL-17 and TNF-α. Astilbin could elevate the Nrf2 accumulation in the nuclei, eventually leading to the transcriptional activation of various antioxidant proteins and reducing the expression of VEGF.

**Conclusions:**

Our results collectively suggest that astilbin could induce Nrf2 nucleus translocation, which is contribute to reduce the ROS accumulation and VEGF expression, and inhibit the proliferation of HaCaT cells.

## Background

Psoriasis is a chronic inflammatory dermatosis that occurs in approximately 2% of adults worldwide [1]. Psoriasis can cause itching with appearing the silvery white scales and raised erythema, and can lead to depressive illness as comorbidity [2], which means patients may suffer both physically and mentally with psoriatic. Psoriasis is also associated with many diseases like hypertension, diabetes mellitus and the metabolic syndrome [3–5]. Apart from the abnormal interconnected networks between immune cells and various inflammatory cytokines, we can also find the pathological features with increased proliferation, aberrant differentiation and impaired apoptosis of keratinocytes in psoriasis lesions [6].

The release of reactive oxygen species (ROS) is regarded as a pro-inflammatory factor, which can induce inflammatory cytokines releasing, DNA modification and oxidative damage [7, 8]. The overproduction of ROS and deficient function of antioxidant system activities are involved in the pathogenesis of psoriasis, therefore alterations of antioxidants are associated with psoriasis and antioxidants are believed to be therapeutic [9–11]. In addition, it is demonstrated that oxidative stress may activate mechanisms that restore the redox balance including activation of the Nrf2, a transcription factor which plays an important role in antioxidant and cyto-protective gene transcription [12, 13]. However, the expression and the activity of Nrf2 in the skin of psoriatic patients were discordant in several studies [10, 14, 15], the mechanisms of Nrf2 regulation in psoriasis are urgently needed to be elucidated.

Astilbin, is a major bioactive compound extracted from Rhizoma Smilacis glabrae (RSG), as a kind of medicinal herb, that is used to treat autoimmune diseases in traditional Chinese medical science [16–18]. Recent researches suggests that astilbin has multiple clinically-relevant bioactivities, including anti-inflammatory [18–20], antioxidant [19, 21], and anti-tumor effects [22]. However, as a well-known antioxidant, the effects and the mechanisms of astilbin on psoriasis remain largely elusive.

The aim of the present study was to investigate the therapeutic efficacy of astilbin in psoriasis. We examined the potential effects of astilbin on ROS accumulation and further demonstrated its correlation with the expression and activation of Nrf2, by using an immortalized human keratinocyte cell line HaCaT as cellular model of psoriasis [23, 24].

## Results

### Astilbin inhibits the proliferation and reduces ROS generation in HaCaT cells

**Fig. 1:**
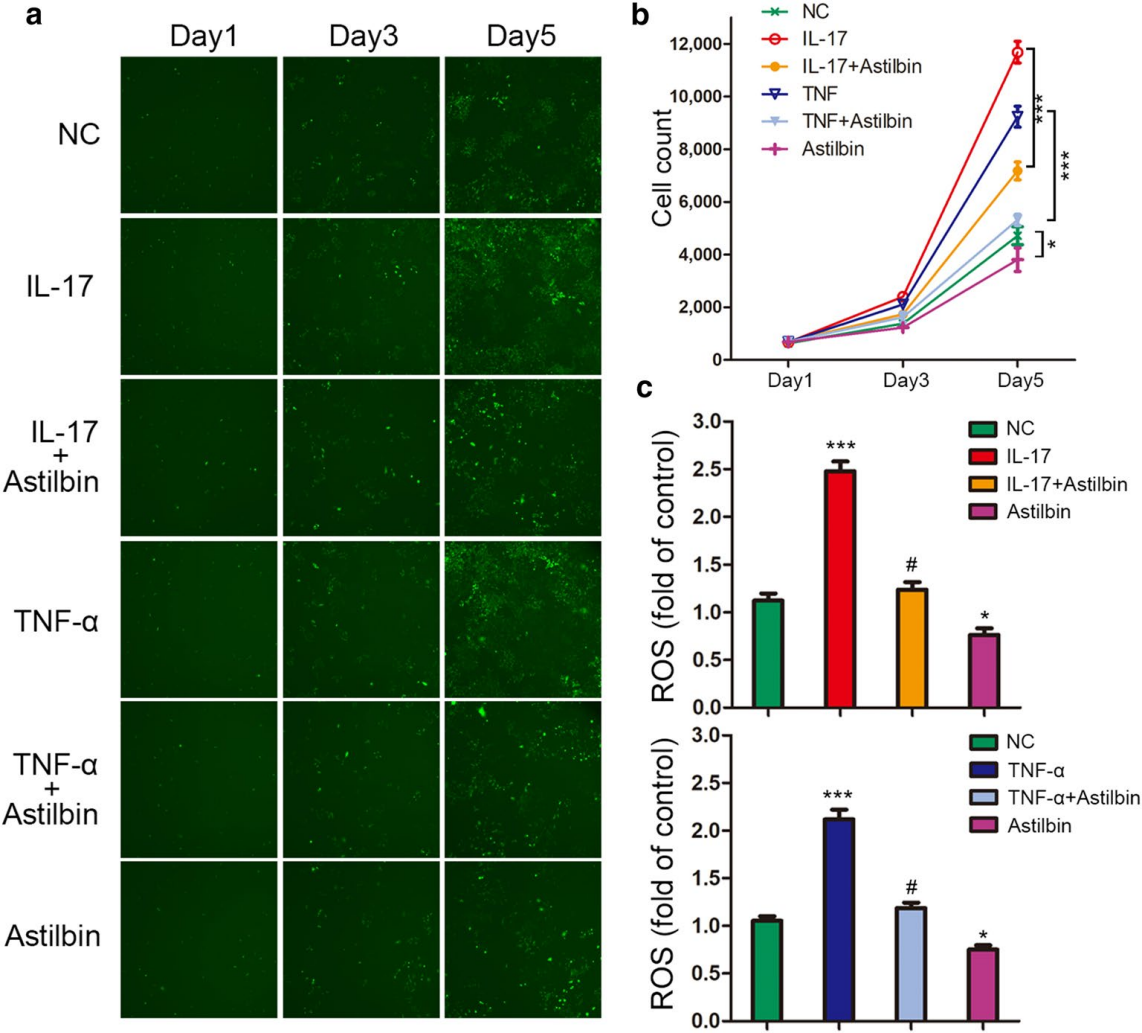
Effects of astilbin on the proliferation and ROS generation in HaCaT cells. **a** Representative pictures of HaCaT cells treated with astilbin and pro-inflammatory cytokines via HCS for day1, day3 and day5. **b** Cell count of HaCaT cells via HCS for day1, day3 and day5. *P < 0.05; ***P < 0.001. **c** Quantification and statistical analysis of ROS-positive cells. *P < 0.05 and ***P < 0.001 vs NC group; ^#^P < 0.05 vs IL-17 or TNF-α group

To investigate the effect of astilbin on cell growth in HaCaT cell lines, we initially stimulated HaCaT cells with IL-17 and TNF-α, the pro-inflammatory cytokines which may contribute to the pathophysiology of psoriasis. Cell growth was monitored by high-content screening (HCS) at day1, day3 and day5. As expected, IL-17 and TNF-α could significantly increase the proliferation of HaCaT cells at day5 (Fig. 1a, b). Treatment with astilbin at 50 μg/ml was introduced at day2, and proliferation changes were observed at day5. The growth curves detected by HCS showed that astilbin treatment dramatically decreased the growth of HaCaT keratinocytes stimulated with IL-17 and TNF-α (P < 0.001), and also in the control group (P < 0.05) (Fig. 1a, b). Since ROS-induced oxidative damage is regarded as a critical mediator in the development of psoriasis, we next examined the effect of astilbin treatment on the accumulation of ROS in HaCaT cells by DCFH-DA staining. As shown in Fig. 1c, IL-17 and TNF-α were able to stimulate ROS generation significantly, however, astilbin treatment resulted in significant reduction of the ROS levels. In addition, astilbin also reduced the ROS production in control group. These results suggested that astilbin could inhibit the growth and reduce the accumulation of ROS in HaCaT keratinocytes stimulated by IL-17 and TNF-α.

### Astilbin enhances Nrf2 activation under pro-inflammatory cytokines stress

**Fig. 2:**
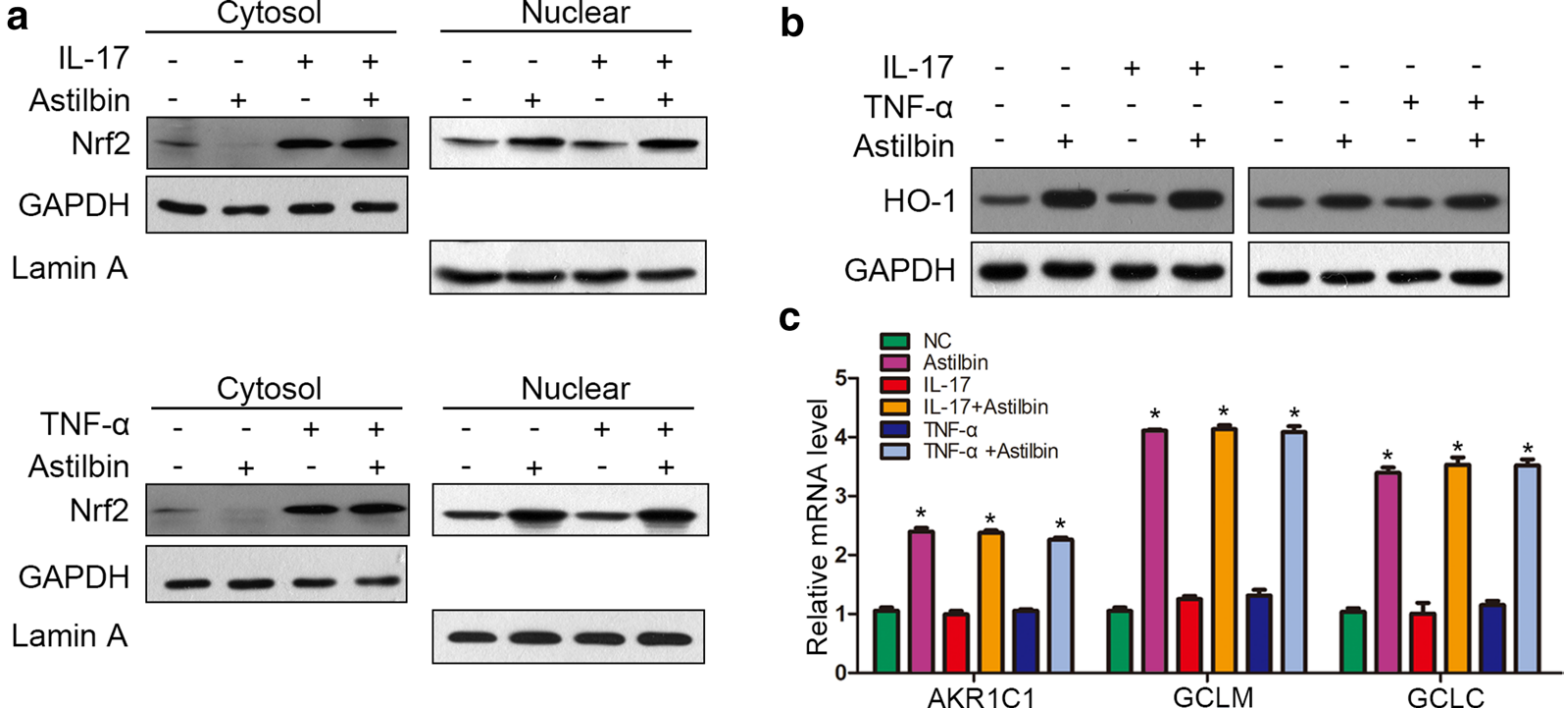
Effects of astilbin on NRF2 activation. **a** The nuclear and cytoplasmic protein expression of NRF2 were determined using Western blot analysis. **b** The expression of HO-1 was determined using Western blot analysis. **c** mRNA levels of AKR1C1, GCLM and GCLC were evaluated using qPCR analysis. *P < 0.05 vs NC group

To identify further the mechanisms of astilbin-exhibited reduction of ROS generation, the expression and the localization of Nrf2 was detected under the stimulation of IL-17 and TNF-α. We found that IL-17 and TNF-α could increase the Nrf2 expression, however, the two cytokines had little effects on the accumulation of Nrf2 in the nuclei. Treatment with astilbin significantly elevated the Nrf2 accumulation in the nuclei and reduce the expression in cytoplasm, which suggests that astilbin may promote the nuclear transfer of Nrf2 (Fig. 2a). In addition, the protein level of HO-1, an antioxidant protein regulated by Nrf2, was significantly up-regulated in the presence of astilbin, regardless of the previous stimulation with proinflammatory cytokine. Next, we examined the mRNA expression of AKR1C1, GCLM and GCLC, which are generally activated by the transcriptional regulation of Nrf2. Our results showed that astilbin increased AKR1C1, GCLM and GCLC mRNA levels in proinflammatory cytokine-stimulated HaCaT cells. These findings demonstrated that astilbin reduced ROS accumulation by enhancing Nrf2 activation.

### Astilbin attenuated the expression of VEGF by Nrf2 activation in HaCaT cells

**Fig. 3:**
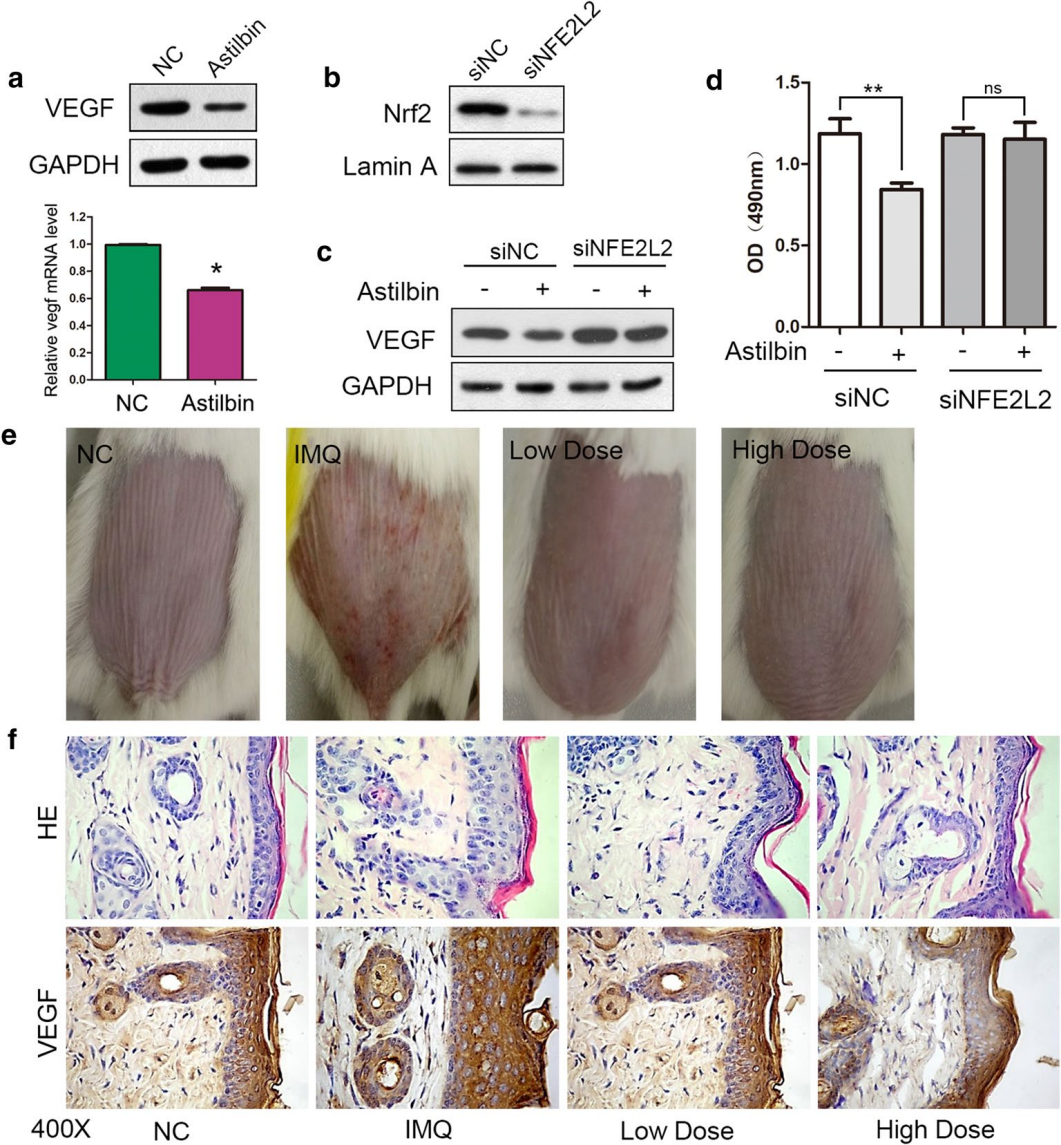
Astilbin attenuated the expression of VEGF. **a** The protein and mRNA level of VEGF were detected by Western blot and qPCR analysis, *P < 0.05 vs NC group. **b** Cells were transfected with 10 nM of si-NFE2L2-B or si-NC, the level of Nrf2 was measured by Western blot analysis. **c** The expression of VEGF were measured by Western blot analysis under treatment with astilbin in Nrf2 knock-down HaCaT cells. **d** MTT assays showing the effect of Nrf2 knock-down on cell viability under astilbin treatment. **P < 0.005; *ns* no significance. **e** The phenotypical presentation of the mice back skin. **f** Psoriasis-like skin lesions were stained with HE, VEGF antibody. *IMQ* imiquimod; Low dose, 10 mg/kg; high dose, 50 mg/kg

Angiogenesis is one of the pathological features of psoriasis. To investigate whether astilbin could have inhibitory effects on angiogenesis, we examined the protein and mRNA levels of vascular endothelial growth factor (VEGF) with astilbin treatment. The results suggested that astilbin significantly decreased the mRNA and protein levels of VEGF (Fig. 3a). We next explored the role of NRF2 in the down-regulation of VEGF by astilbin. Three pairs of NFE2L2-siRNA were used to knock down Nrf2 expression in HaCaT cells. Western blot analysis showed that siNFE2L2-B could effectively silence the expression of Nrf2 (Fig. 3b). Then, we analyzed protein levels of VEGF in Nrf2 knockdown HaCaT cells. We found that astibilin did not altered VEGF expression in NRf2 knockdown HaCaT cells (Fig. 3c). Furthermore, Nrf2 inhibition annulled the inhibitory effect of astilbin on the proliferation of HaCaT cells (Fig. 3d).

To investigate whether astilbin has a beneficial effect on psoriasis, we administered astilbin to a mouse model of psoriasis. Topical application of imiquimod (IMQ) was used to establish the model. One intragastric administration of astibilin was introduced in two groups: one with low dose (astibilin 10 mg/kg), and one with high dose (50 mg/kg astilbin). The severity of psoriasis was determined according to the PASI (the Psoriasis Area and Severity Index) score at day20 (Table 1) and the phenotypical presentation photos of the mice back skin are shown in Fig. 3e. HE staining revealed that IMQ increased epidermal hyperplasia, acanthosis in the epidermis and perivascular infiltration of the inflammatory cells in the upper dermis, which corresponds with the pathological characteristic of psoriasis lesions. However, the lesions significantly resolved with astilbin administration, which was corroborated by a decrease in the thickness of the epidermis layer, which attenuated IMQ-induced psoriasis (Fig. 3f). We also detected the expression of VEGF in the skin. Immunohistochemical staining for VEGF also revealed that that administration of astilbin decreased the number of VEGF positive cells in IMQ-induced psoriasis (Fig. 3f and Table 2). Taken together, these results demonstrate that administration of astilbin efficiently suppresses the expression of VEGF in vivo and vitro, probably through enhancing NRF2 activation.

**Table 1:** PASI score at day 20

Group	Erythema n = 10	Induration n = 10	Desquamation n = 10	PASI
NC	0	0	0	0
IMQ	2.2 ± 0.42	2.1 ± 0.32	2.2 ± 0.42	6.5 ± 0.71*
Low dose	1.8 ± 0.42	1.7 ± 0.48	1.8 ± 0.42	5.3 ± 0.48^#^
High dose	1.5 ± 0.53	1.4 ± 0.52	1.5 ± 0.53	4.4 ± 0.70^#^

* P < 0.05 compared with NC group

^#^P < 0.05 compared with IMQ group

**Table 2:** VEGF expression in IMQ-induced psoriasis

Item	NC	IMQ	Low dose	High dose
	n = 10	n = 10	n = 10	n = 10
VEGF	0.942 ± 0.087	6.329 ± 0.437*	4.112 ± 0.259^#^	1.307 ± 0.124^#,^^

* P < 0.05 compared with NC group

^#^P < 0.05 compared with IMQ group

^^^P < 0.05 compared with low dose group

## Discussion

Psoriasis is a chronic skin disease characterized by scaling, thickening and erythema of the skin, which is probably the most prevalent immune-mediated skin disease in adults [25]. Recent studies show that some mutations may lead to an abnormally increased activity of the immune system and, consequently, to increased inflammation and accompanying oxidative stress [26]. The endogenous antioxidant defence system of the body fails to replenish the damage, and the unfavourable skin metabolism further worsens the situation for patients with psoriasis [7]. It has also been suggested that generation of ROS from keratinocytes and fibroblasts can contribute to neutrophil activation, which plays an important role in psoriatic process [27, 28].

It is reported that astilbin from *Smilax glabra Roxb.* had protective effect on lead-induced oxidative stress [29], so we speculated that the antioxidant effect of astilbin could ameliorate the progression of psoriasis. Consequently, our results demonstrated that astilbin could reduce ROS generation and inhibit the proliferation of HaCaT cells. In addition, astilbin could elevate the Nrf2 accumulation in the nuclei to enhance its transcription factor activity, eventually leading to the transcriptional activation of various antioxidant proteins and even reducing the expression of VEGF. Therefore, we suggest that antioxidants, like astibilin, might act as a new therapeutic approach in psoriasis.

Nrf2, a transcription factor of the cap “n” collar family, is a crucial regulator involved in cellular resistance to oxidants [30]. Intracellular ROS levels are regulated by an inducible antioxidant program that responds to cellular stressors and is largely correlated with the activation of Nrf2, which regulates the expression of multiple cytoprotective genes to counteract oxidative stress [31, 32]. Altered antioxidant response of Nrf2 increases the susceptibility to chemically induced skin cancer [33], and the inflammation of the skin lesion would be prolonged when its antioxidant effect is impaired [34]. Therefore, targeting the regulation of NRF2 activation can be contribute to prevent the risk of oxidative stress-related skin lesion. A recent study demonstrated that natural flavonoids can increase the expression of protective genes through activation of Nrf2. Although the effect of astilbin on Nrf2 in cisplatin-induced acute nephrotoxicity model has been reported [35], the underlying mechanism was still unknown. In this study, we explored the effect of astilbin on ROS accumulation and found astilbin strongly induced Nrf2 nucleus translocation to stimulate the expression of Nrf2-mediated antioxidant genes in HaCaT cells, suggesting that increasing the activation of Nrf2 may contribute to antioxidant effect of astilbin on keratinocyte cells line.

Vessel hyperplasia is one of the characteristics of psoriatic lesions. Vascular endothelial growth factor (VEGF) can be secreted by keratinocytes and is assumed to be involved in the pathogenesis of psoriasis [36]. Recent evidence indicated that the chronic administration of VEGF to the skin can result in development of psoriasis-like inflammation [37]. In the present study, we found that astilbin could reduce the expression of VEGF. However, astilbin was ineffective when Nrf2 was silenced, suggesting that astibilin effect in VEGF expression is dependent of Nrf2 activation and nuclear translocation.

## Conclusions

In conclusion, our data suggested that astilbin could reduce the ROS accumulation and down-regulate the expression of VEGF by inducing Nrf2 nucleus translocation, which finally contributes to reduce the proliferation of HaCaT cells. Future studies may focus on the molecular mechanisms by which astilbin inhibits inflammation through Nrf2 activation.

## Method

### Cell lines and treatment

The HaCaT cell line were obtained from the Cell Bank of Type Culture Collection of Chinese Academy of Sciences (Shanghai, China). HaCaT cells were cultured in Dulbecco’s Minimum Essential Medium supplemented with 10% fetal bovine serum at 37 °C in a humidified atmosphere containing 5% CO_2_. All of the culture media were purchased from Sigma (St. Louis, MO, USA), and fetal bovine serum was purchased from Gibco (catalogue no. 16000-044; Grand Island, NY, USA). IL-17A and TNF-α were purchased from Abcam (IL-17A, ab9567; TNF-α, ab9642). Cells were cultured to 80% confluence and incubated with IL-17A (50 ng/ml) or TNF-α (10 ng/ml) for 24 h. Astilbin was purchased from Ourchem Sinopharm Chemical Reagent (CAS#: 29838-67-3, XW080058) and was dissolved in dimethyl sulfoxide (DMSO, Beyotime, ST038) at a concentration of 10 mM and stored at − 20 °C.

### High-content screening cell proliferation assay

Cell growth was measured via multiparametric high-content screening (HCS). After 72 h of infection with the NC lentivirus, HaCaT cells were re-suspended, counted and inoculated into 96-well plates. With the treatment of astilbin for 1 day, 3 day and 5 day, the proliferation of the cells in each of the wells was analysed daily using Metaxpress HCS software (Molecular Devices). The system uses a computerized, automated fluorescence imaging microscope that automatically identifies stained cells and measures the intensity and distribution of fluorescence in each individual cell.

### ROS detection

Intracellular ROS levels were evaluated by 2′,7′-dichlorodihydrofluorescein diacetate (DCFH-DA, Sigma Aldrich, D6883) staining assay, the HaCaT cells were incubated with DCFH-DA (10 μM) according to the manufacturer’s instructions. The fluorescence was measured using flow cytometry (C6, Millipore).

### Western blotting

Cytoplasmic and nuclear extracts were prepared according to the instructions of the NE-PER Nuclear and Cytoplasmic Extraction kit (Thermo Scientific, 78833). Samples from cell lysates were separated by SDS-polyacrylamide gel electrophoresis, transferred onto polyvinylidene difluoride membranes, and incubated with primary antibodies, including: Nrf2 (1:1000; Abcam, ab137550), HO-1 (1:1000; Abcam, ab68477), VEGF (1:1000; Proteintech, 19003-1-AP), Lemin A (1:1000; Cell Signaling, 86846s), and GAPDH (1:2000; Cell Signaling, 5174s), followed by incubation with horseradish peroxidase (HRP)-conjugated secondary antibody (1:2000; Cell Signaling, 7074v/70). Protein expression was visualized by enhanced chemiluminescence assay.

### Quantitative real-time PCR

Total RNA was isolated from frozen tissues and cell lines using TRIzol (Invitrogen, USA), according to the manufacturer’s instructions. RNA was used for the first-strand cDNA synthesis with a Takara RNA PCR Kit (Takara). Real-time PCR was performed using SYBR Green Premix Ex Taq Ver. 3.0 (Takara) and detected by StepOne plus. GAPDH was used as an internal control. The primers used were as follows: AKR1C1 forward, 5′-TGGGAGGCCGTGGAGAA-3′; AKR1C1 reverse, 5′-GGACACCCCGATGGACCTG-3′; GCLM forward, 5′-ACAGGTAAAACCAAATAGTAACCAAGTTAA-3′; GCLM reverse, 5′-TGTTTAGCAAATGCAGTCAAATCTG-3′; GCLC forward, 5′-GATGCTGTCTTGCAGGGAATG-3′; GCLC reverse, 5′-AGCGAGCTCCGTGCTGTT-3′; GAPDH forward, 5′-AAGGTCGGAGTCAACGGATTTG-3′; and GAPDH reverse, 5′-CCATGGGTGGAATCATATTGGAA-3′.

### siRNAs and transfection

The targeting sequence for Nrf2 siRNA was: NFE2L2-B, 5′-AAUUCCAAGUCCAUCAUGCUG-3′ (sense), 5′-GCAUGAUGGACUUGGAAUUGC-3′ (antisense). Transfection of siRNA into HaCaT cells was carried out using Lipofectamine 2000 (Invitrogen, Carlsbad, CA, USA) following the manufacturer’s instructions.

### Mouse studies

Six-week-old male BALB/c mice were purchased from Changchun Yisi Laboratory Animal Center. All experimental procedures involving mice were in compliance with the National Institutes of Health Guide for the Care and Use of Laboratory Animals and were approved by the Review Committee for the Use of Animals of Inner Mongolia University for Nationalities. Mice were topically treated with IMQ cream (China Meheco Keyi Pharma Co., Ltd, H20040285) on the shaved back skin (2 cm * 2 cm) once a day for 7 days to induce a psoriasis-like dermatitis. Different doses of astilbin were selected for intragastric administration at day7, once a day for 13 days. The mice were sacrificed after 20 days, and the back skin were harvested. According to the PASI scoring standard, erythema (redness), induration (thickness) and desquamation (scaling) are scored as 0 to 4 in the skin lesions, and intensity scores are added up for the total score. The intensity of redness, thickness and scaling of the psoriasis is assessed as none (0), mild (1), moderate (2), severe (3) or very severe (4).

### Immunohistochemistry

Standard protocols were used to prepare tissue microarray sections for immunohistochemistry. Then sections were blocked by UltraVision Hydrogen Peroxide Block (Thermo Scientific, CA, USA) and UltraVision Protein Block (Thermo Scientific), followed by primary antibodies (VEGF, 1:100) incubation. UltraVision Quanto Detection System horseradish peroxidase (HRP) Polymer (Thermo Scientific) and DAB Quanto (Thermo Scientific) were applied for staining, and hematoxylin was used for counterstaining. The score of erythema, scale and infiltration thickening of the skin lesions in mice was scored as 0 to 4: 0, negative; 1, weak; 2, moderate; 3, strong; and 4 very strong. Add the three scores to get the total points, taking the average of the scores of the mice in each group, and skin lesions in each group were observed.

### Statistical analysis

Analysis was performed with SPSS software, version 22.0 (SPSS, Chicago, IL, USA). The data are presented as the mean ± standard deviation, with at least three replicates used for each data point. Differences between two groups were analyzed with Student’s t test. Statistical significance was determined at the level of P < 0.05.

## Data Availability

All data generated or analysed during this study are included in this published article.

## Abbreviations

ROS: reactive oxygen species
VEGF: vascular endothelial growth factor
Nrf2 or NFE2L2: Nuclear factor (erythroid-derived 2)-like 2
IL-17: interleukin-17
TNF-α: tumour necrosis factor α
HCS: high-content screening
DCFH-DA: 2′,7′-dichlorodihydrofluorescein diacetate
HO-1: heme oxygenase 1
AKR1C1: Aldo-keto reductase family 1 member C1
GCLM: glutamate–cysteine ligase regulatory subunit
GCLC: glutamate–cysteine ligase catalytic subunit
IMQ: imiquimod
